# An outbreak of gastroenteritis by emerging norovirus GII.2[P16] in a kindergarten in Kota Kinabalu, Malaysian Borneo

**DOI:** 10.1038/s41598-020-64148-4

**Published:** 2020-04-28

**Authors:** Kamruddin Ahmed, Jiloris Julian Frederick Dony, Daisuke Mori, Liaw Yun Haw, Nelbon Giloi, Mohammad Saffree Jeffree, Hidekatsu Iha

**Affiliations:** 1 Borneo Medical and Health Research Centre, Faculty of Medicine and Health Sciences, Universiti Malaysia Sabah, Kota Kinabalu, 88400 Sabah Malaysia; 2 Department of Pathobiology and Medical Diagnostics, Faculty of Medicine and Health Sciences, Universiti Malaysia Sabah, Kota Kinabalu, 88400 Sabah Malaysia; 3 Public Health Laboratory, Sabah State Health Department, Kota Kinabalu, 88300 Sabah Malaysia; 4 KPJ Sabah Specialist Hospital, Kota Kinabalu, 88300 Sabah Malaysia; 5 Department of Community Medicine and Public Health, Faculty of Medicine and Health Sciences, Universiti Malaysia Sabah, Kota Kinabalu, 88400 Sabah Malaysia; 6 Department of Microbiology, Faculty of Medicine, Oita University, Yufu, 879-5593 Oita Japan

**Keywords:** Viral epidemiology, Viral transmission

## Abstract

Outbreaks of diarrhea in kindergartens are underreported and frequently go unnoticed in developing countries. To better understand the etiology this study was performed during an outbreak of diarrhea in a kindergarten in Sabah, Malaysia. Outbreak investigation was performed according to the standard procedures. In this outbreak a total of 34 (36.5%) children and 4 (30.8%) teachers suffered from gastroenteritis. Stool samples from seven children and 13 teachers were tested for rotavirus and norovirus. During the investigation stool samples were collected and sent in cold chain to the laboratory. The samples were subjected to rotavirus enzyme linked immunosorbent assay, and reverse transcription PCR for norovirus. All samples were negative for rotavirus but positive for norovirus. To determine the genogroup and genotype of norovirus, nucleotide sequencing of the amplicons was performed. All norovirus from the outbreak was of genotype GII.2[16]. To determine the relatedness of the strains phylogenetic analysis was done using neighbor-joining method. Phylogenetically these strains were highly related to GII.2[P16] noroviruses from China and Japan. This study provided evidence that a diarrheal outbreak in a kindergarten was caused by GII.2[P16] norovirus which is an emerging strain in East Asia and Europe.

## Introduction

Norovirus is highly infectious and is transmitted primarily from person-to-person through the fecal-oral route within closed settings such as schools, cruise ships, hospitals, childcare facilities and aged-care institutions^1,2^. Currently this virus is divided into ten genogroups GI–GX based on the genetic differences of the capsid protein^3^. GI and GII are primarily responsible for human infection^4^. GII.4 causes most infections worldwide, followed by GII.3 or GII.6 and then other genotypes in varying proportions^5–10^. Although norovirus infection is an important cause of diarrhea in developing countries, outbreaks are under reported and frequently go unnoticed. In Kota Kinabalu, the capital of Sabah state in Malaysia, only two diarrheal outbreaks, including this one, have been reported in kindergartens between 2014 and June 2018. However, kindergartens are vulnerable to norovirus outbreaks: in Pudong district, Shanghai, China, 29 of 60 reported norovirus outbreaks occurred in kindergartens over a period of one year^11^. Therefore, understanding the epidemic situation, relationship with other strains, evolution, genetic variants and the genotype distribution of norovirus during outbreaks is important to determine the best strains to include in future norovirus vaccines. Global surveillance data indicates that several norovirus genotypes are responsible for outbreak but majority are caused by GII.4^12,13^. Since 2002, new GII.4 variants have emerged every two to three years, resulting in epidemics and global pandemics^13^. To emerge and persist in human population norovirus generate diverse strains by point mutations and recombination^14^. However, recent outbreaks of norovirus particularly in Asia and Europe were caused by GII.2[P16] and GII.17^15–20^. Therefore, the purpose of the present study was to determine the etiology and genetic characterization of the causative agent of a diarrheal outbreak occurred among kindergarten children in Kota Kinabalu.

## Methods

Upon information from a private hospital of several children with gastroenteritis from a particular kindergarten during February 2017, a team from the Kota Kinabalu Health Office started an investigation according to the standard procedures of the Malaysian Ministry of Health. An outbreak was declared on February 23, 2017. During the investigation, recent gastroenteritis could be documented in 11 children and four teachers. Stool collection tubes were distributed on March 1, 2017 and stool samples from seven children and 13 teachers were available for investigation. The samples were sent to University Malaysia Sabah in cold chain for virus detection.

Phosphate-buffered solution was added to stool samples to a final concentration of 10% (mass/volume). The samples were briefly vortexed, followed by centrifugation at 12,000 rpm for 1 min and supernatant was collected. Supernatant was used for rotavirus detection by ELISA and RNA extraction.

A commercial ELISA kit (Rotaclone, Meridian Diagnostics, Cincinnati, OH, USA) was used to detect rotavirus in stool samples according to manufacturer’s instructions. RNA from fecal samples was extracted using the QIAamp Viral RNA Kit (QIAGEN, Hilden, Germany). Norovirus was detected by reverse transcription (RT)–PCR by amplifying the capsid gene at the C region^21^. The amplicon size of the partial capsid gene of GI and GII noroviruses are 330 and 344 bp, respectively. The RNA-dependent RNA polymerase (RdRp) gene was amplified by RT-PCR^22^. The amplicon size of the partial RdRp gene is 470 bp. The RT–PCR results were confirmed by nucleotide sequencing of the amplicons^5^. The nucleotide sequence of the amplicons was determined by the BigDye Terminator v3.1 Cycle Sequencing Kit (Applied Biosystems, Foster City, CA) according to the manufacturer’s instructions and the product was run on a ABI Prism 3100 Genetic Analyzer (Applied Biosystems). The genogroups and genotypes were determined by submitting nucleotide sequences to the Norovirus Genotyping Tool (http://www.rivm.nl/mpf/norovirus/typingtool).

Partial nucleotide sequences of the capsid gene at C region (262 nt in length) and RdRp (331 nt in length) were used for phylogenetic analyses. The nucleotide sequences of other GII.P16 and GII.2 noroviruses were extracted from GenBank. Multiple sequence alignment was carried out using ClustalW, and a phylogenetic tree was constructed using MEGA 7.0, applying the neighbor-joining method based on the Tamura–Nei substitution model^23^. Bootstrap analysis of 1,000 replicates was conducted to determine the significance of the branching of the constructed tree.

To compare the pathogen distribution with that of sporadic gastroenteritis cases among children, we tested rotavirus and norovirus in 20 stool samples collected from children under 5 years of age who were admitted from August 15, 2016 through May 26, 2017 in a private tertiary care hospital in Kota Kinabalu with diarrhea.

The study was registered, and ethical clearance was obtained from the National Medical Research Register (NMRR-18-2646-44291) and the ethical committee of the Faculty of Medicine and Health Sciences, Universiti Malaysia Sabah. All experiments were performed in accordance with the relevant guidelines and regulations. Informed consents were obtained from teachers and guardians of the children.

## Results

The onset of gastroenteritis in the index case was on February 21, 2017. The last case appeared on February 27, 2017. During the outbreak, there were 106 contacts in the kindergarten, of whom 93 were children and 13 teachers. The mean age of the children was 4 years 6 months (range 2 years 2 months–6 years 1 month). The male-to-female ratio was 1.02:1. All teachers were female with a mean age of 30.5 years (range 18–55 years). A total of 34 (36.5%) children and 4 (30.8%) teachers suffered from gastroenteritis, which represents an attack rate of 35.8%. The kindergarten contained six classes, and the distribution of infected cases among the total number of children in each class was as follows: 6/15 (40.0%), 5/17 (29.5%), 6/17 (35.3%), 6/13 (46.1%), 5/13 (38.5%) and 6/18 (33.3%). The primary case was a 4-year-old girl; teachers noted that she had gastroenteritis when kindergarten opened soon after the Chinese New Year, which ended on January 29, 2017. Subsequently, new cases started to appear as diarrhea spread among the children and staff.

All stool samples were tested negative for rotavirus but norovirus was detected in stool samples from one teacher and four children. The genotype of noroviruses identified was GII.2[P16].

**Figure 1:**
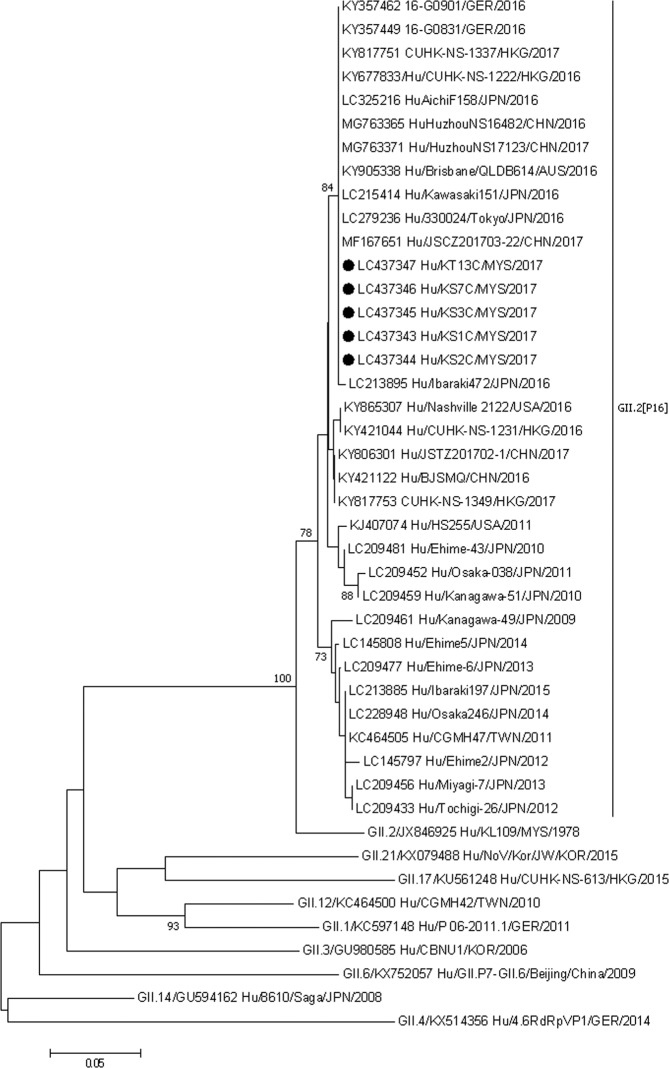
Phylogenetic tree constructed using the partial nucleotide sequences of the capsid protein genes of norovirus strains applying the neighbor-joining method based on the Tamura–Nei substitution model. The strains analyzed in this study are marked with a filled circle. The number adjacent to the node represents the bootstrap value. Values <70% are not shown. The scale bar at the bottom indicates the genetic distance expressed as nucleotide substitutions per site. The nucleotide sequences of our strains have been submitted to the databases of the DNA DataBank of Japan, the European Molecular Biology Laboratory, and GenBank with accession nos. LC437343 – LC437347.

**Figure 2:**
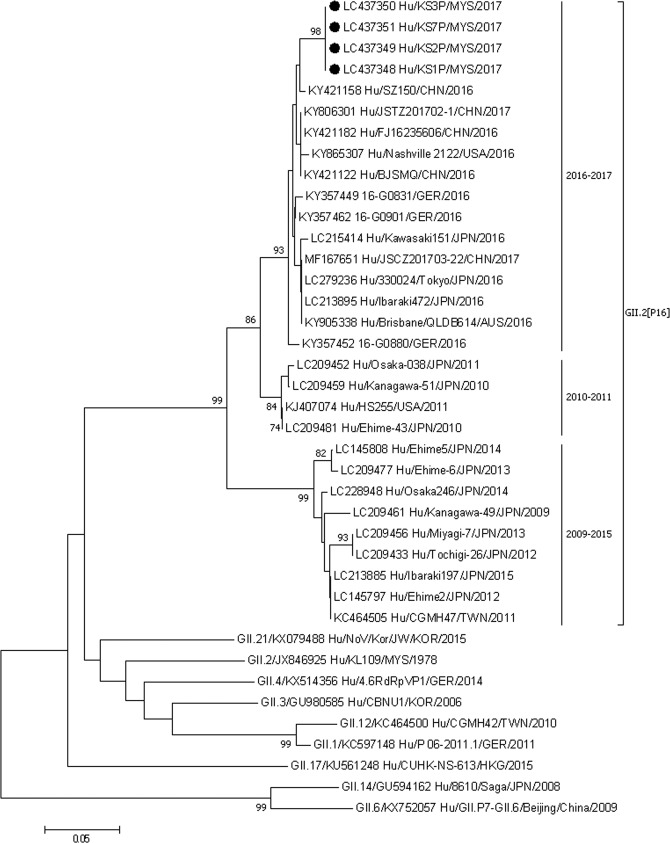
Phylogenetic tree constructed using the partial nucleotide sequences of the polymerase genes of norovirus strains applying the neighbor-joining method based on the Tamura–Nei substitution model. The strains analyzed in this study are marked with a filled circle. The number adjacent to the node represents the bootstrap value. Values <70% are not shown. The scale bar at the bottom indicates the genetic distance expressed as nucleotide substitutions per site. The nucleotide sequences of our strains have been submitted to the databases of DNA DataBank of Japan, the European Molecular Biology Laboratory, and GenBank with accession nos. LC437348– LC437351.

The phylogenetic analysis of the capsid gene (Fig. 1) showed that our strains formed a cluster that had a significant bootstrap value (>70%) with the 2016–17 GII.2[P16] norovirus outbreak strains from Tokyo, Kanagawa and Ibaraki prefectures of Japan, Zhejiang and Jiangsu provinces of China, Germany, sporadic cases from Hong Kong in 2017 and Aichi prefecture of Japan. The capsid genes of our strains all showed 100% nucleotide identity and except for one strain from Japan (Ibaraki 472), our strains had 99.6% nucleotide identity with other strains of the cluster. In the phylogenetic tree of the RdRp gene our strains formed a distinct subcluster within the cluster of 2016–17 GII.2[P16] norovirus strains from Japan, China, Germany and Australia which were in the cluster of capsid gene phylogenetic tree (Fig. 2). This cluster also contains norovirus strains from Nashville, China and Japan that did not cluster with our strains in the capsid gene phylogenetic tree. The RdRp genes of our strains were all 100% identical and showed 97.0–97.9% identity with other strains of the cluster.

Of 20 stool samples collected from the private hospital, 13 (65%) contained rotavirus, 4 (20%) contained norovirus of genotype GII.4[P16]. Neither rotavirus nor norovirus was detected in the remaining 3 (15%) samples.

## Discussion

Since the GII.2[P16] strains from Kota Kinabalu, formed cluster with strains from China, Japan, Germany, and Australia in the phylogenetic trees constructed by using the capsid and RdRp genes therefore it is indicating that these strains have the same ancestor. However, the strains from Kota Kinabalu formed a subcluster in the RdRp gene phylogenetic tree which is possibly indicating few nucleotide substitutions in that gene. Although the RdRp gene has undergone few nucleotide substitutions, the capsid gene has remained unchanged, possibly because the capsid gene of GII.2 exhibits only limited antigenic evolution^24^. The GII.2[P16] strains of norovirus have been increasingly reported from Asia such as China, Japan, Hong Kong, Taiwan, and Thailand during the 2016–2017 season^18,20,25,26^. The GII.2[P16] strains have been also reported from Europe such as Germany, Italy, and France^17,20,27^. Although the GII.2[P16] genotype has been reported from some sporadic cases, it is predominantly associated with outbreaks^15–20^. In China, 79% of the 56 norovirus outbreaks were caused by GII.2[P16] strains and 38 (78%) of GII.2 outbreaks occurred in kindergartens^28^. This indicates that possibly kindergarten children are vulnerable to GII.2[P16] strains, however more evidences are needed to confirm this. Outbreaks of norovirus infection are difficult to control that authorities are forced to close schools, hospital wards and food outlets, which lead to severe economic and social consequences^1^. One of the limitations of this study was our inability to trace the source of the outbreak because the children were too young to remember whether they had had contact with another patient out of school or had consumed food suspected to be contaminated^29^. However, norovirus outbreaks transmitted by ingestion of contaminated foods such as oysters are generally caused by several genotypes^2,30^, while norovirus spreads by person-to-person transmission in semi-closed communities is usually traced to a single genotype^2^. In the present study the detection of only GII.2[P16] genotype of norovirus from stool samples, 100% sequence homology of the capsid and RdRp genes, and the continuation of infections spread for several days indicates that it was spread by person-to-person transmission. The symptoms of norovirus infection last between 24 and 72 h, people may continue to shed virus for several weeks after symptom resolution^1,31^ which might be one of the factors for the spread of infection from apparently recovered children. Another limitation is the delay in notification by the relevant hospital. Thus, less samples were collected than the number of people documented with diarrhea during the initial investigation and lack of data to provide an epidemiological curve of cases.

The circulating genotype of noroviruses responsible for sporadic cases of diarrhea in Kota Kinabalu was GII.4[P16] whereas the genotype associated with the outbreak was GII.2[P16]. Worldwide norovirus GII.4 is the predominant cause of sporadic gastroenteritis^32^. The polymerase genotype associated with GII.4 varies, recently GII.4[P16] has been emerged in many industrialized countries^13,33^. Further study is needed to determine the distribution of GII.4[P16] in Kota Kinabalu and their relationship with recently emerged GII.4[P16] strains in other countries.

In conclusion we determined an outbreak in a kindergarten in Sabah caused by the emerging GII.2[P16] strains which is prevalent in East Asia and Europe. Sabah is considered as a remote place, however, our study demonstrated that in today’s world of connectivity no place is spared from emerging pathogens. Studies on norovirus are sparse in Malaysia and the burden of this virus is unknown among its population as a result norovirus is not generally considered for outbreak investigation^3^. This is the first study in Malaysia where we have characterized the causative norovirus up to genotype level. Further studies are needed to determine the extent of norovirus outbreaks and their genotype distribution in Malaysia.
